# Chemopreventive Effect of Dietary Anthocyanins against Gastrointestinal Cancers: A Review of Recent Advances and Perspectives

**DOI:** 10.3390/ijms21186555

**Published:** 2020-09-08

**Authors:** K.V. Surangi Dharmawansa, David W. Hoskin, H. P. Vasantha Rupasinghe

**Affiliations:** 1 Department of Plant, Food, and Environmental Sciences, Faculty of Agriculture, Dalhousie University, Truro, NS B2N 5E3, Canada; surangi@dal.ca; 2 Department of Pathology, Faculty of Medicine, Dalhousie University, Halifax, NS B3H 4R2, Canada; D.W.Hoskin@dal.ca; 3 Department of Microbiology and Immunology, and Department of Surgery, Faculty of Medicine, Dalhousie University, Halifax, NS B3H 4R2, Canada

**Keywords:** polyphenols, bioavailability, gastrointestinal tract, inflammation, chemoprevention, molecular mechanisms

## Abstract

Anthocyanins are a group of dietary polyphenols, abundant mainly in fruits and their products. Dietary interventions of anthocyanins are being studied extensively related to the prevention of gastrointestinal (GI) cancer, among many other chronic disorders. This review summarizes the hereditary and non-hereditary characteristics of GI cancers, chemistry, and bioavailability of anthocyanins, and the most recent findings of anthocyanin in GI cancer prevention through modulating cellular signaling pathways. GI cancer-preventive attributes of anthocyanins are primarily due to their antioxidative, anti-inflammatory, and anti-proliferative properties, and their ability to regulate gene expression and metabolic pathways, as well as induce the apoptosis of cancer cells.

## 1. Introduction

The term “cancer” is described as a sequence of complex processes involving the accumulation of altered genetic material in cells, unlimited cell proliferation, and the formation of malignant tumors, cells from which can then migrate to and invade distant sites of the body [1]. According to the World Health Organization (WHO), cancer is responsible for one in six deaths worldwide, causing about 30% of all premature deaths in adults aged 30–69 years. Despite improvements in therapeutic strategies and screening programs, in 2018, 18.1 million people had cancer worldwide and WHO forecasts doubling of cancer cases by 2040 [2]. Among all types of cancer, gastrointestinal (GI) cancers, which include cancers of the colon and rectum (colorectal), esophagus and stomach (gastroesophageal), liver, gallbladder, pancreas, small intestine, appendix, and anus, collectively represent one of the greatest public health problems in the world, accounting for more than 35% of cancer-related deaths [2]. GI cancers have common risk factors; however, GI cancers are different in etiological, epidemiological, and clinical management profiles [3]. Colorectal cancer has become the third most common cancer in the world, and all other GI cancers still add a burden to the global incidence of cancer due to the limited number of biomarkers available for cancer screening, diagnosis, and prognosis [4]. Each year, approximately 4.1 million people are diagnosed with GI cancers, and about 3 million cancer-related deaths are due to late detection of the disease [5].

The carcinogenesis of GI cancers is linked to several molecular abnormalities, which include and are not limited to epigenetic modifications such as DNA methylation [6], and inactivation of tumor suppressor genes, i.e., TP53, which results in irregular cell cycle replication processes [7], and activation of oncogenes and various telomerases [8]. Moreover, the imbalance between cell proliferation and apoptosis leads to the pathogenesis of GI cancers [7]. Internal factors, such as chronic inflammation, which is influenced by the intestinal microbial imbalance, promote the malignant transformation of healthy cells into cancerous cells [9]. However, a third of all cancers are due to unsatisfactory lifestyles and dietary practices [10]. Alcohol consumption and exposure to environmental pollutants promote GI cancer, while regular consumption of plant-based foods containing dietary fiber reduces the risk of GI cancer [11].

Flavonoids, a group of C15 polyphenols, have been the subject of extensive research for their potential in chemoprevention and chemotherapy [12]. Flavonoids are abundant in berries (blueberry, raspberry, haskap berry, blackberry, and elderberry), vegetables (broccoli, kale, lettuce, and celery), tea, coffee, and red wine [13,14,15]. Flavonoids have gained attention as anticancer agents due to their structural diversity, relative abundance, limited toxicity, and cancer-preventive efficacy [16]. Among the major sub-groups of flavonoids, anthocyanins are widely found in plant-based food, including more than 1000 water-soluble compounds responsible for the vivid blue, purple, and red nuances of fruits, vegetables, colored grains, and beans [16,17,18].

Cancer chemoprevention refers to the use of agents for the inhibition, delay, or reversal of carcinogenesis before the local invasion of tissues occurs [19]. The results of epidemiological studies suggest that anthocyanins inhibit the initiation and progression of GI cancers [20]. The underlying molecular mechanism of anthocyanins and their colonic microorganism-generated metabolites in chemoprevention has been attributed to their antioxidant potential, anti-inflammatory activity, anti-proliferation effect, induction of apoptosis and suppression of matrix metalloproteinases in cancer cells [21]. In addition, anthocyanins are capable of stimulating the expression of tumor suppressor genes and downregulating pro-oncogenic signals [22]. The present review summarizes the latest findings on the potential of anthocyanin in the prevention of GI cancers, as well as their underlying molecular mechanisms of action, as evidenced by in vitro, in vivo, pre-clinical, and clinical studies.

## 2. GI Carcinogenesis

GI cancers account for more than 20% of cancers worldwide. Even countries with a high standard of living, education, and health experience a high incidence of GI cancers and associated morbidity and mortality [23]. GI cancer is a heterogeneous cancer that tends to occur in the more common sporadic forms rather than the rare inherited forms. The process of initiation and formation of neoplastic cells in the GI tract can be classified into four main mechanisms: (i) inherited transmission of mutations; (ii) exposure to different carcinogens; (iii) chronic inflammatory conditions/microbial dysbiosis; and (iv) sporadic mutations and epigenetic changes [24].

### 2.1. Hereditary GI Cancers

**Figure 1:**
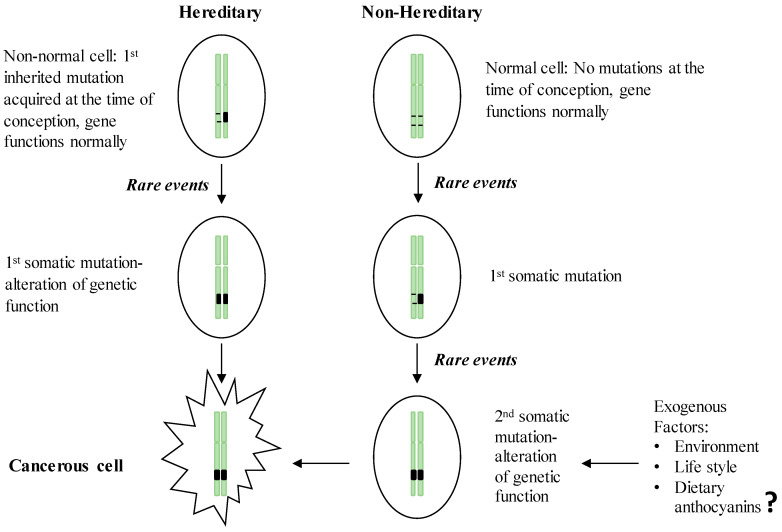
Two-hit theory of the initiation of hereditary and non-hereditary cancer. People with a hereditary susceptibility to GI cancers harbor an inherited genetic mutation on one of the chromosomes at the time of conception and receive the 1st somatic mutation due to the endogenous (e.g., chronic inflammation) or exogenous (e.g., exposure to carcinogens) rare events which in turn inactivate the full function of the respective gene and initiate neoplastic transformation. Non-inherited forms of GI cancer occur by acquiring two somatic mutations in later life, resulting in the inactivation of a gene leading to the initiation of malignancy.

Hereditary GI cancers represent a phenotypically diverse group of diseases involving malignant tumors of the digestive tract, extra-GI cancers, and benign abnormalities characterized by inherited genetic mutations transmitted from parent to child. However, no more than 3%–5% of GI cancers have shown a clear hereditary basis [25]. The esophagus, stomach, colon, small intestine, and pancreas have been identified as the organs most likely to inherit germline mutations [24]. The best known inherited malignant tumors are associated with the GI tract, representing monogenic hereditary diseases that result from mutations in a single gene [26]. Despite the specific differences in the genes involved, inherited GI cancers share a common set of characteristics: (i) the majority of GI cancers are detectable in the early stages of life; (ii) these cancers follow an autosomal dominant inheritance mechanism in which the neoplasm occurs in 1st degree relatives; and (iii) the formation of multiple tumors [26,27]. In the hereditary form of GI cancers, the first genetic mutation in one of the alleles of a predisposition gene is acquired at the time of conception, and the somatic mutation of the second allele is then acquired via environmental insult, lifestyle practices or other exogenous factors (Figure 1). Once the two alleles of a specific predisposition gene are mutated, gene function is completely inactivated, leading to carcinogenesis. Compared to the sporadic form of GI cancer, which requires two somatic events during the inactivation of the predisposition gene, hereditary cancers present a higher risk because they need only one somatic mutation event, which explains the early onset of hereditary cancers [28]. In parallel with the advancement of DNA technologies, the genetic mutations responsible for hereditary GI cancers have been widely documented (Table 1). These hereditary GI cancers include Cowden syndrome, MUTYH-associated polyposis, hereditary pancreatic cancer, Lynch syndrome, Peutz-Jeghers syndrome, familial adenomatous polyposis (FAP), attenuated FAP, serrated polyposis syndrome, and hereditary gastric cancer. Cancer-causing mutations can be initiated in three main classes of predisposition genes, oncogenes, tumor suppressor genes, and DNA repair genes, which are involved in establishing genetic stability.

### 2.2. Non-Hereditary GI Cancers

Accumulations of sporadic mutations can occur due to factors such as exposure to carcinogens [24], a westernized diet [42,43], diets rich in salt [44,45], obesity [46,47], chronic alcohol consumption [48,49], and chronic inflammation [50]. The relationships between carcinogens, diet, inflammation, and GI cancers are multiple and complex. Exposure to carcinogens can initiate cancer development via somatic mutations that include point mutations, deletions, additions, and modified methylation of DNA [51]. There are several cellular mechanisms to protect DNA from carcinogen-induced mutations and to identify and correct these mutations before they give rise to malignancy. In spite of these protective mechanisms, the GI tract is continuously exposed to chemical and biological carcinogens, often due to diets that act as carriers of preformed carcinogens [24]. Among known carcinogens, tobacco smoke hydrocarbons are one of the most potent, being comprised of more than 60 mutagens and cancer-causing chemicals directly linked to esophageal [51], pancreatic [52], and gastric cancers [53,54]. In addition, exposure to airborne occupational carcinogens such as cement dust, quartz dust, and diesel exhaust fumes increases the risk of gastric cancer [55]. Nitrosamines, which are produced from the chemical reaction between nitrates or, in reduced form, nitrites with amines present in meat products during the meat preservation process, are another group of potent carcinogens associated with the increased risk of malignancy in the liver and GI tract due to DNA alkylation and DNA adduct formation [56]. Among biological carcinogens, aflatoxin B1 is one of the most influential hepatocarcinogens produced by the *Aspergillus flavis* fungi. Due to its lipophilic nature, aflatoxin B1 is readily absorbed from the GI tract. Aflatoxin B1, upon its metabolism by cytochrome P450 in the liver, induces irreversible mutations in the p53 gene of hepatocytes [57,58]. Fumonisin B1 is another mycotoxin that can cause hepatic and esophageal cancers [59]. Fumonisin B1 acts in part by upregulating the production of inflammatory cytokines by gastric and colon epithelial cells [60].

GI tract carcinogenesis is attributed to chronic inflammatory conditions that occur due to microbial, viral, or disease conditions such as inflammatory bowel disease (IBD). Regarding microbial inflammation, *Helicobacter pylori* infection is well documented as a trigger for GI cancers. Exposure to *H. pylori* initiates active chronic gastritis by increasing the infiltration of inflammatory cells, which leads to the formation of intestinal adenocarcinoma and other malignant tumors of the GI tract [61]. In addition, hepatitis B and C virus infection, gastroesophageal reflux, enzyme damage, autoimmune diseases such as ulcerative colitis, and systemic stress conditions are responsible for chronic GI inflammation [24]. Clinical studies show that patients with IBD have a significantly higher risk of developing colorectal cancer, especially 8 to 10 years after the diagnosis of IBD [62]. The mechanisms underlining the link between inflammation and GI cancers are varied and include the production of high levels of reactive oxygen species (ROS) and reactive nitrogen intermediates (RNI) [63]. The macrophages that dominate the chronic inflammatory microenvironment produce increased levels of ROS and RNI, which in turn interact with the DNA of proliferating epithelial cells and generate permanent genetic mutations leading to the malignant transformation. Excessive ROS/RNI production during the process of oxidative metabolism has been reported to promote the synthesis and secretion of inflammation-promoting cytokines such as tumor necrosis factor (TNF)-α, interferon-gamma (IFN-γ), and interleukin (IL)-6 [64,65]. Moreover, other inflammatory mediators such as chemokines, growth factors, and eicosanoids in tumor microenvironments contribute to inflammation-triggered tumor progression and metastasis via modulating the immune response, inhibiting apoptosis, inducing cell proliferation, and promoting the accumulation of oncogenic mutations [66].

Diet, personal lifestyle, and the environment are all linked to the development of GI cancers. Chronic excessive caloric intake and physical inactivity leading to overweight and obesity-derived metabolic dysfunction are essential risk factors of GI carcinogenesis [46,47]. Energy imbalance causes alterations in glycemic control, insulin signaling, and upregulation of adipose tissue-derived inflammatory pathways that prolong carcinogenesis-promoting conditions [67]. Chronic alcohol consumption also increases susceptibility to GI cancers [48]. Acetaldehyde, the primary metabolite of alcohol, has recently been targeted for its involvement in ethanol-linked oxidative stress and the inhibition of DNA methylation by interfering with the metabolism of B vitamins, reducing the activity of methionine synthase and glutathione levels [68], and disrupting retinoid metabolism [48]. Depletion of systemic and tissue-specific retinoic acid levels are associated with possible malignant transformation; thus, chronic alcohol consumption reduces hepatic vitamin A and retinoic acid levels, which are strongly related to the later development of hepatocellular carcinoma (HCC) via decreasing mitogen-activated protein kinase (MAPK) and increasing levels of phosphorylated c-Jun N-terminal kinases (JNKs) [48]. Moreover, in vivo evidence reveals that high-fat and high-salt diets alter the permeability and growth of the colonic mucus layer, which in turn, leads to intestinal microbial dysbiosis that is linked with an increased incidence of GI cancer [69]. Colonic microbial imbalance resulting from prolonged consumption of westernized diets enhances the breakdown and metabolism of specific glycans in the mucus layer, leading to GI carcinogenesis [70].

Among the modifiable risk factors of GI tract cancers, diet has been identified as one of the most significant in cancer control. Extensive studies have shown the chemopreventive effect of dietary polyphenols [71,72]. These natural antioxidants can prevent the onset of GI tract cancers, thus enhancing human well-being [73]. Polyphenols are potent scavengers of ROS and other free radicals that cause DNA damage and neoplastic transformation [74]. Chemopreventive effects of polyphenols extend to the prevention of pro-carcinogen activation, downregulation of inflammation, and inhibition of cell proliferation by interfering with the cell cycle activities of cancer cells [75]. Anthocyanins, a sub-class of flavonoids categorized under the group of polyphenols, have also become prominent dietary antioxidants in GI cancer prevention owing to their strong electron donor ability [76]. According to cohort studies, frequent consumption of fruits and vegetables of vivid blue, purple and violet colors, the richest sources of anthocyanins, have been associated with a reduction in the incidence of colorectal cancer [77], bladder cancer [78], and gastric cancer [79].

## 3. Chemistry, Dietary Sources, Bioavailability, and Toxicology of Anthocyanin

**Figure 2:**
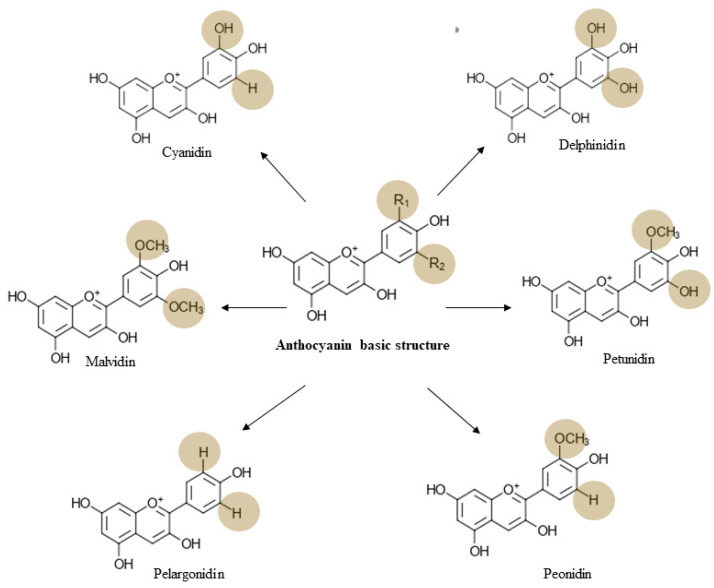
Major anthocyanins derived from the basic anthocyanin structure. Based on the changes in R_1_ and R_2_ chemical groups, six major anthocyanins have been identified.

Anthocyanins, which are a glycosidic form of anthocyanidins, possess a basic structure of C6-C3-C6 composed of two aromatic rings (A and C) and one heterocyclic ring (B) [80] (Figure 2). Anthocyanins are differentiated on the basis of the number of hydroxyl groups, the number and type of sugar moieties, and the presence or absence of acyl groups [16]. Out of over 600 anthocyanins identified in nature, six main anthocyanin classes are well distributed in fruits and vegetables (Figure 2). Cyanidin-3-*O*-glucoside (C3G) is highly abundant among anthocyanins, and more than 90% of anthocyanins are conjugated with glucose [81]. Families of Vitaceae (grape), Rosaceae (cherry, plum, raspberry, strawberry, blackberry, apple, and peach), Solanaceae (tamarillo and eggplant), Saxifragaceae (red and black currant), Caprifoliaceae (haskap), Cruciferae (red cabbage) and Ericaceae (blueberry and cranberry) are primary sources of dietary anthocyanin [20,82]. Due to their anionic nature, once consumed, anthocyanins undergo pH and physiological temperature-dependent transformations that have a significant impact on their biological activities, improving their capacity to mediate cancer chemoprevention [73]. Despite the beneficial properties and relative abundance of anthocyanins, their effectiveness in the prevention of cancers depends on their bioavailability. Intact forms of anthocyanins that are absorbed from the stomach, as well as the intestine via an active transport mechanism, are then subject to hepatic Phase 2 metabolism. The resulting anthocyanin metabolites enter the systemic circulation. Unabsorbed anthocyanins reach the large intestine and undergo microbial biotransformation into decomposed products that contribute to cancer-chemoprevention [83]. Gastric digestion does not significantly affect anthocyanin composition; however, approximately 42–76% of total anthocyanins and 29% of their antioxidative activity are lost during passage through the intestines [84,85]. A ^13^C traceability study that utilized eight healthy male participants revealed 12% relative bioavailability of C3G after receiving a 500 mg oral dose of anthocyanin [86]. In contrast, a recent human intervention study showed that only 0.02% of ingested bilberry anthocyanin is detectable in plasma over 8 h after ingestion [87]. These controversial findings indicate that further investigations of bioavailability, absorption, and excretion of anthocyanins are warranted. The maximum plasma concentration is attained within 0.5–2 h after the consumption of anthocyanin-rich foods [83]. Around 20–25% of the ingested anthocyanin is absorbed by the gastric mucosa, although this varies according to the structure of the anthocyanin [86,87,88,89]. The majority of glycosidic forms, anthocyanin monoglucosides, and non-acylated compounds are well absorbed [90,91]. Glucose transporters are not involved in gastric absorption of anthocyanin; hence, absorption is facilitated by bilitranslocase, an organic anion membrane carrier [92]. Unabsorbed anthocyanin is then metabolized into glucuronidate, sulfate, or methyl derivatives in the small intestine; the greatest amount is absorbed in the jejunum and the lowest is absorbed by duodenal tissue [93]. Anthocyanins that pass down to the large intestine are subjected to spontaneous or microbial bioconversion [94]. In vitro studies prove that upon bacterial metabolism, cleavage of glycosidic linkage and breakdown of the anthocyanidin heterocycle is possible while producing 4-hydroxybenzoic acid, protocatechuic acid (PCA), gallic acid, vanillic acid, and syringic acid as the major microbial metabolites [95]. Incubation of a mixture of anthocyanins with fecal bacteria results in the formation of gallic, syringic, and p-coumaric acids [96]. The metabolism of C3G and cyanidin-3-*O*-rutinoside by rat gut microflora gives rise to protocatechuic, vanillic, p-coumaric acids, and 2,4,6-trihydroxybenzaldehyde. Gallic acid, syringic acid, and 2,4,6-trihydroxybenzaldehyde are the primary metabolites of delphinidin-3-*O*-rutinoside [97]. Therefore, microbial metabolism of anthocyanins may contribute to their pronounced chemopreventive properties, as the microbiome enhances anthocyanin metabolite concentrations [98].

Consumption of anthocyanins has been generally considered as safe in humans and anthocyanin consumption has been increased in line with educational level and degree of physical activity of populations [99]. As far as we are aware, there are no adverse health issues reported concerning anthocyanin in reported human intervention studies. Usually, the doses used in dietary supplementations of anthocyanin are higher than the regular dietary intakes.

## 4. Mechanisms of Anthocyanin-Mediated Chemoprevention of GI Cancers

**Figure 3:**
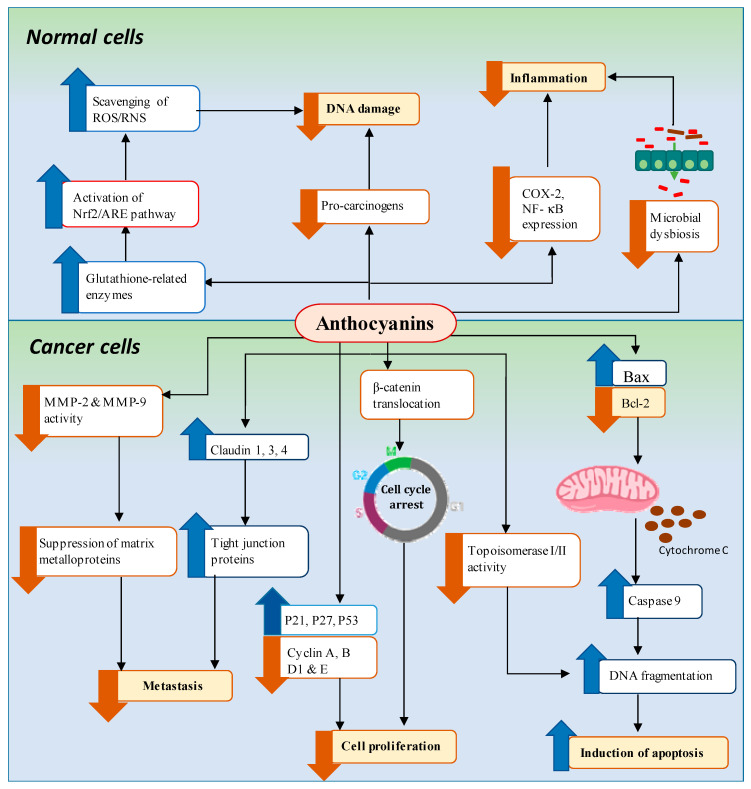
The possible anticarcinogenic mechanisms of anthocyanins in GI cancer prevention. Anthocyanins inhibit the pro-inflammatory COX-2 and NF-κB pathways and inhibit cell proliferation via reducing the nuclear translocation of β-catenin, upregulating cyclin-dependent kinase inhibitors, and downregulating cyclin proteins. Anthocyanins reduce the degradation of components of the extracellular matrix by suppressing the activity of MMPs and tight junction (TJ) proteins. Anthocyanins act as topoisomerase inhibitors and stimulate the DNA strand break response, leading to apoptosis. Anthocyanins induce apoptosis via the mitochondrial pathway and activation of caspase-9. Anthocyanins modulate gut microbial dysbiosis, hence reducing the production of ROS in macrophages and suppressing chronic inflammation. P21, P27, P53, cyclin-dependent kinase inhibitors; COX-2, cyclooxygenase-2; NF-κB, nuclear factor κ-light-chain-enhancer of activated B cells; MMP-2 and 9, matrix metalloproteinases 2 and 9; ROS, reactive oxygen species. Blue upwards arrow, promote; orange downwards arrow, inhibit.

The mechanisms by which anthocyanins prevent GI cancers are not well understood. However, anthocyanins have emerged as promising chemopreventive compounds for GI cancers, most likely because of their antioxidant, anti-inflammatory, anti-cell proliferative, and apoptosis-inducing properties [21]. A recent study demonstrates that anthocyanins reduce carcinogen-induced DNA damage in cultured human lung epithelial cells [100], pepsin-induced DNA damage in human airway epithelial cells [101], and benzo-[a,1]-pyrene dihydrodiol (DBP-diol)-induced DNA adducts and DBP-diol and DBP-diolepoxide (DBPDE)-induced mutagenesis in lacI rat oral fibroblast cells and human oral leukoplakia cells [102]. Extensive investigations have been performed to determine the molecular mechanisms underlying the chemopreventive properties of anthocyanins. The results indicate that anthocyanins inhibit several signaling pathways involved in DNA damage, cancer initiation, cancer cell proliferation, and tumor growth [20]. Potential molecular mechanisms of anthocyanin-mediated GI cancer prevention are summarized in Figure 3.

### 4.1. Downregulation of Pro-Inflammation and Oxidative Stress Associated with DNA Damage

#### 4.1.1. Pro-Inflammation

Chronic inflammation is a prolonged immune response that contributes to the pathogenesis of GI cancers [103]. Under chronic inflammatory conditions, intestinal barrier function is impaired by the loss of the mucosal epithelial layer integrity layer due to decreased production and assembly of the TJ proteins and translocation of invasive microbial species and microbial products to the internal tissue environment [104]. In various systems, anthocyanins improve the intestinal TJ barrier integrity by promoting the expression of crucial barrier-forming TJ proteins such as occludin, claudin-5 and, zonnula occuldin-1 via upregulation of glucagon-like peptide (GLP)-2 intestinal hormone levels [105,106]. In addition, anthocyanins tend to improve barrier function by regulating TJ and epithelial cell permeability [107]. Anthocyanins also down-regulate the expression of major pro-inflammatory biomarkers such as TNF-α, IL-6, IL-1β, IFN-γ, prostaglandin E2 (PGE2), monocyte chemoattractant protein (MCP)-1, cyclooxygenase (COX) -2, and nuclear factor kappa B (NF-κB) [108,109,110,111]. For example, a combination of lycopene and anthocyanin inhibits expression of the cytokine IL-8, whereas, anthocyanin-rich wild blueberry extract reduces the activity of NF-κB in Caco-2 cells [111,112]. Anthocyanins extracted from red clover [113], and black rice [114], inhibit the translocation of NF-κB into the nucleus of lipopolysaccharide (LPS)-activated RAW264.7 macrophages. Furthermore, the production of nitric oxide (NO), expression of COX-2 and secretion of TNF-α and IL-6 were also diminished by black rice extracts [114]. Overexpression of the pro-inflammatory enzyme, inducible nitric oxide synthase (iNOS), is another general feature of epithelial tissue inflammation and carcinoma development [115]. In this regard, Peng et al. [116] report that the long term consumption of anthocyanin from *Lycium ruthenicum* Murray reduces inflammation of the colon by reducing the expression of iNos, Cox-2, TNF-α, IL-6, IL-1β, and IFN-γ mRNAs in C57BL/6 male mice. Additionally, cocoplum extract, which is rich in the anthocyanins delphinidin, cyanidin, petunidin, and peonidin, downregulates IL-1β, IL-6, and NF-κB expression in HT-29 colorectal adenocarcinoma cells while decreasing TNF-α-induced intracellular ROS-production [117]. Moreover, anthocyanins from various sources, for example, fruits of *L. ruthenicum* Murray [109], red raspberry [118], black rice [119], and strawberry [120], are able to attenuate dextran sulfate sodium (DSS)-induced gut inflammation in mouse models of IBD. Thus, suppression of inflammation by anthocyanins may protect against GI cancer occurrence or its progression.

#### 4.1.2. Oxidative Stress Associated with DNA Damage

During chronic inflammation, excessive production of ROS and RNI leads to a disruption of redox homeostasis, producing oxidative stress. The redox homeostasis imbalance causes direct cellular damage by oxidation of macromolecules, including oxidative DNA damage resulting in DNA mutations [121]. Moreover, oxidative stress contributes to cancer progression by continuously creating DNA mutations in cancerous cell populations. Modulation of intracellular oxidative stress by scavenging the ROS/RNI is, therefore, beneficial in preventing cancer initiation and progression [122]. Interestingly, anthocyanins are potent inhibitors of redox dysregulation due to their ability to increase the oxygen radical-absorbing capacity of intestinal cells [123], stimulate phase II detoxification enzymes [124], reduce the formation of oxidative DNA adducts, and decrease lipid peroxidation [125]. For example, redox homeostasis in Caco-2 and HT-29 cells is restored by bilberry extract, which reduces intracellular ROS production and oxidative DNA damage, as well as increasing cellular glutathione-s-transferase (GSH) levels [126]. The antioxidant potential of anthocyanins has also been investigated in artificial alimentary tract models, including models of the stomach, small intestine, and colon. A digested form of anthocyanin extracted from purple carrot was effective in reducing oxidative DNA damage in colon mucosa and inhibited intracellular ROS while modulating the oxidative imbalance in rat liver induced by cadmium exposure [127,128]. In another study, digested products of wild raspberry, including primarily esculin, kaempferol hexoside, and pelargonidin hexoside, displayed a more pronounced effect against acrylamide-induced cytotoxicity in Caco-2 cells in comparison to the non-digested extract, which was related to reduced ROS generation and GSH depletion [129]. The antioxidative and anti-inflammatory effects of anthocyanins in intestinal ischemia-reperfusion (IIR) injury have also been reported [130,131]. Dietary supplementation with chokeberry and bilberry alone or together with probiotics inhibits oxidative stress and tissue injuries in mouse models of IIR [130]. However, anthocyanin supplements are unable to bring about a significant reduction in oxidative stress markers and pathology associated with DSS-induced colitis in Balb/c mice [132]. These occasionally inconsistent and somewhat variable results indicate that validation is required for the role of anthocyanins in modulating inflammation and oxidative stress. Nevertheless, anthocyanin-mediated prevention of hepatocarcinogenesis via activation of the Nrf-2/ARE pathway is well documented. For example, blackcurrant anthocyanins protect against diethylnitrosamine (DENA)-initiated hepatocarcinogenesis in rats by elevating the expression of protein and mRNA related to the Nrf-2 pathway [133]. These examples further support the notion that dietary anthocyanins play a significant role in the chemoprevention of colitis-associated GI cancer.

### 4.2. Inhibition of Cancer Cell Proliferation/Induction of Cell Cycle Arrest

The cell cycle consists of a programmed sequence of events beginning with cell size increase (G_1_ phase), DNA replication (S phase), cell preparation (G_2_ phase), and finally, cell division (M phase), which are coupled with G_1_-S, S, and G_2_-M checkpoints [134]. Under normal physiological conditions, cell cycle progression is governed by the activation/inactivation of cyclins and cyclin-dependent kinases (CDKs) [135]. The G_1_-S and S phases of the cell cycle are mainly regulated by CDK_4_-cyclin D, CDK_6_-cyclin D, CDK_2_-cyclin E, and CDK_2_-cyclin A sequential complexes while G_2_/M is controlled by CDK_1_-cyclin A/B [136]. Upon the segregation of DNA mutations, CDK inhibitors (CDKIs) such as p21 (cip1/waf1/cap20/sdi1/pic1), p27 (kip1), p57 (kip2) specific for CDK_2_ and CDK_4_ cyclin complexes and p16^INK4^, p15^INK4B^, p18^INK4C^ and, p19^INK4D^ specific for CDK_4_ and CDK_6_ cyclin complexes bind to and inactivate their respective CDK-cyclin complexes, thereby blocking cell cycle progression [137]. Hence, dysregulation of the cell cycle often leads to aberrant cell proliferation, which results in malignant cell growth during which loss of control of cell cycle checkpoints results in genetic instability [138]. Concerning GI cancers, anthocyanins prevent cancer by initiating cell cycle arrest at various stages and inducing anti-proliferative activity in a dose-dependent manner [115,139,140]. Anthocyanins are capable of upregulating CDKIs and downregulating cyclin proteins [141]. An anthocyanin-rich extract of chokeberry shows anti-proliferative effects resulting from cell cycle arrest at both G_1_/G_0_ and G_2_/M phases in HT-29 human colon cancer cells due to upregulation of p21, p27, and downregulation of cyclin A and B [142]. Consistently, anthocyanin metabolites, gallic acid, 3-*O*-methyl gallic acid, and 2,4,6-tri-hydro benzaldehyde, show the ability to block the proliferation of Caco-2 cells at G_0_/G_1_ phase [22]. Anthocyanins are also potent inducers of cell cycle blockage at the G_2_/M phase in oral cancer KB cells by down-regulating p53 methylation [143]. In addition to stimulating the expression of p21 and p27 CDKIs, an anthocyanin/anthocyanidin-rich extract from purple shoot tea reduced cyclin E and cyclin D1 expression in HT-29 colorectal carcinoma cells, resulting in cell cycle arrest at G_0_/G_1_ phase [144]. Similarly, delphinidin prevents HCT-116 cell proliferation by blocking the G_2_/M phase due to underexpression of cyclin B1 and overexpression of p53, a tumor suppressor protein, and p21^WAF1/cip1^ [145]. However, different anthocyanins have different effects related to cell cycle control. For example, chokeberry anthocyanins that consist mainly of cyanidin derivatives are more potent inhibitors of HT-29 cell proliferation than grape or bilberry anthocyanins, which are rich in delphinidin [146]. Similarly, malvidin and pelargonidin (100 to 200 µL/mL) effectively suppress stomach and colon cancer cell proliferation, which is not affected by cyanidin and delphinidin [147]. Both peonidin-3-glucoside and C3G interfere with CDK-1,2 and cyclin B1 expression in AGS-gastric adenocarcinoma and SKHep-1, Huh-7 hepatocellular carcinoma cells. However, the activity of cyclin E expression is only inhibited by peonidin-3-glucoside, whereas only C3G inhibits cyclin E1 expression [148].

### 4.3. Induction of Apoptosis

Cells with damaged or mutation-containing DNA are normally eliminated by a form of cell death known as apoptosis [149]. Two distinct but interacting pathways mediate apoptosis; the extrinsic (death receptor-mediated) pathway, which activates caspase-8, and the intrinsic (mitochondrial membrane-permeabilizing) pathway, which activates caspase 9. Caspases are aspartate-specific cysteine proteins that stimulate nuclear membrane degradation, chromatin condensation, DNA fragmentation, and the formation of apoptotic bodies [150]. However, cancerous cells often fail to undergo apoptosis and, therefore, survive to form a tumor. In some cases, cancer cells may resist apoptosis by increasing or decreasing expression of anti- or pro-apoptotic genes, respectively. Moreover, cancer cells may also prevent apoptosis by changing the functions of anti- or pro-apoptotic proteins through post-translational modifications, such as phosphorylation [151].

Anthocyanins can activate both extrinsic and intrinsic pathways of apoptosis. Mechanisms of action include upregulating the expression of pro-apoptotic proteins such as B-cell lymphoma-2-like protein 4 (Bax) while downregulating the expression of anti-apoptotic proteins such as B-cell lymphoma-2 (Bcl-2), X-linked inhibitor of apoptosis protein (XIAP), caspase-recruitment domains like apoptotic proteins (CIAP)-1,2 and survivin [151,152]. For example, anthocyanins reduce the expression of anti-apoptotic proteins, survivin, CIAP-2, and XIAP in HT-29 and HCT-116 human colon carcinoma cells [153]. Human hepatoma Hep3B cells treated with an anthocyanin-rich extract from meoru (*Vitis coignetiae Pulliat*) exhibited significantly reduced Bcl-2, XIAP, and CIAP 1,2 protein expression [154]. Furthermore, anthocyanins increase DNA fragmentation, as indicated by the number of cells in the sub-G_1_ fraction, in a dose-dependent manner, which is closely related to mitochondrial dysfunction. The mitochondrial pathway of apoptosis is characterized by a profound reduction in mitochondrial membrane potential (ΔΨm). The collapse of ΔΨm leads to the opening of mitochondrial permeability transition pores in the mitochondrial membrane, thus allowing the release of cytochrome C into the cytosol, which in turn triggers caspase-9 activation and the ensuing irreversible events of the apoptosis cascade [155]. Interestingly, anthocyanins induce ΔΨm loss in GI carcinoma cells [156]. In gastric cancer cells, malvidin promotes an increase in Bax/Bcl-2 ratio, caspase-3 activation, and p38 kinase expression while reducing the ΔΨm and inducing cell cycle blockage at the G_0_/G_1_ stage [157]. Similarly, Yun et al. [145] report the cleavage of poly(ADP)-ribose polymerase (PARP), activation of caspases-3, -8, and -9, reduction of Bcl-2, and increased Bax protein expression in HCT-116 human colon cancer cells treated with anthocyanins. Although changes in Bcl-2 mRNA expression are not certain in HT-29 cells treated with bilberry extract, the pro-apoptosis marker, Bax, was increased 1.3-fold in cloudberry and bilberry treated cells [158]. The degree of cell growth inhibition followed the sequence bilberry > black currant > cloudberry > lingonberry > raspberry > strawberry, emphasizing the effect of divergence in anthocyanin source on potential GI cancer prevention. This may be due to variations in the anthocyanin profile of these fruits.

Topoisomerase inhibitors are efficient inducers of apoptosis [159]. Topoisomerase I and II enzymes play a vital role in DNA replication, facilitating the unwinding of supercoiled DNA. Inhibition of topoisomerase activity, therefore, prevents DNA replication, leading to apoptosis [160]. Anthocyanins are naturally occurring topoisomerase inhibitors [161]. Topoisomerase relaxation activity is inhibited by anthocyanin from blackberry extract at a concentration > 50 µM in the colon tissue of male Wistar rats [162]. In a similar study, berry extract at > 50 µM suppressed the activity of topoisomerase I in HT-29 cells while diminishing the activity of topoisomerase II at concentrations ≥ 1 µM [163]. However, concentrations up to 50 µM failed to induce DNA strand breaks. In contrast, C3G-rich blackberry extract suppressed camptothecin (CPT)- or doxorubicin (DOX)-induced stabilization of the covalent DNA-topoisomerase intermediate in HT-29 colon carcinoma cells [164]. These results, taken together, suggest that anthocyanins induce apoptosis in GI carcinoma cells in a dose-dependent manner via activation of extrinsic and intrinsic pathways of apoptosis, as well as by interfering with topoisomerase activity.

### 4.4. Regulation of Microbial Dysbiosis

Accumulating evidence indicates both a negative and positive association between gut microbiota and GI cancers. Healthy gut bacteria or probiotics are involved in activating anti-tumor immunity and boosting the efficacy of immunotherapy, whereas harmful bacteria induce inflammation-driven DNA alterations [165]. Anthocyanins and gut microbiota exhibit a two-way interaction that impacts host physiology. There is a broad agreement that dietary anthocyanins are involved in the modulation of gut microbiota, increasing the ratio of healthy/unhealthy bacteria [104]. For example, oral administration of 5 g/kg body weight of black raspberry to the diet for six weeks resulted in an increase in the abundance of healthy microbial species such as *Akkermansia* and *Disulfovibrio* (known to have anti-inflammatory effects) in F-344 rats [166]. C57BL/6J mice with colitis that were fed malvidin-3-glucoside at a dose of 24 g/kg body weight also showed a decrease in the number of pathogenic bacteria such as *Ruminococcus gnavus*, thereby restoring the gut microbial balance [167]. In a recent study, oral gavage of malvidin-3-galactoside (40–80 mg/kg body weight) increased butyric-producing bacteria and reduced the abundance of pathogenic bacteria in C57BL/6J mice with liver carcinogenesis [168]. As pathogenic microbes are involved in intestinal inflammation, regulation of gut microbial composition by anthocyanin is directly linked with the reduction of inflammation, hence preventing the onset of GI carcinogenesis. However, as the evidence of anthocyanins in microbial modulation is limited, additional research should be carried out to fully elucidate these interactions.

## 5. Anti-GI Cancer Effect of Common Dietary Anthocyanins

Although there is regular global consumption of a wide source of dietary anthocyanins, the scope of this review is limited to the chemopreventive effects of anthocyanin-rich fruits and cereals against cancers of the GI tract (Table 2). Selected major GI-cancers will be discussed in relation to the effect of anthocyanins based on in vitro and pre-clinical studies. Epidemiological studies will be discussed separately.

### 5.1. Oral Cancer

Malignancies that arise on the lips, tongue, gingiva, mouth floor, parotid, and salivary glands are defined as squamous cell carcinomas (SCC) or oral cancers [200]. Chemoprevention with anthocyanins may be useful for oral carcinomas as clinicians and patients can directly monitor the premalignant lesions, and medications can be applied directly to the affected area. Anthocyanins have been incorporated into bio-adhesive gels for the purpose of oral cancer prevention by inhibiting the malignant transformation of dysplastic oral lesions [143]. Intraoral bioactivation of anthocyanin occurs through the activities of oral microflora and salivary enzymes via β-glucosidase activity [152]. The anti-cancer activity of anthocyanins towards SCC is based on several factors: (1) Individual variations in anthocyanins uptake and intraoral metabolism; (2) pH dependency of the mucoadhesive gel on the penetrability of the anthocyanins, and (3) sustainability of anthocyanins at the target site [201]. As an example, berry gels prepared using 5% and 10% *w/w* freeze-dried black raspberry powder are absorbed readily into human oral mucosa tissue within five minutes and show more significant penetrability at pH 6.5 [202]. Studies with human oral epidermal KB and SCC131 cells show that anthocyanins induce significant apoptosis and cell cycle arrest at G_2_/M and G_1_/S phases, respectively [143]. Anthocyanins have also been shown to suppress the metastasis of human tongue epithelial CAL 27 cells and oral SCC cells [170,171]. In contrast, anthocyanin-rich cranberry extract is less able to suppress the growth of SCC of tongue and KB cells compared to the positive control drug Adriamycin [169]. As there is limited evidence on the anti-cancer effects of anthocyanins on oral cell carcinoma, additional studies are needed to determine more precisely the chemopreventive effects of these natural source compounds.

### 5.2. Esophageal Cancer

Due to its aggressive nature and poor survivability, esophageal cancer is the 10th most common malignancy and the 8th leading cause of cancer-related deaths worldwide [203]. Alcohol, tobacco, hot beverage, and red meat consumption are implicated as risk factors for the two prominent types of esophageal cancers, esophageal squamous cell carcinoma (ESCC), and adenocarcinoma [174,204]. A recently published meta-analysis of epidemiological studies reveals a positive correlation between anthocyanin intake and reduced esophageal cancer risk [203]. Anthocyanins are capable of reducing markers of inflammation and angiogenesis and inhibiting the migration and proliferation of human esophageal microvascular endothelial cells isolated from donor-discarded esophagus [174]. Reduced COX-2 and iNOS expression is observed in RE-149DHD and RE-149 rat esophageal cancer cell lines treated with freeze-dried black raspberries [175]. Fisher 344 (F344) rats are used extensively to model esophageal cancer induced by the nitrosamine carcinogen, N-nitroso methylbenzylamine (NMBA) [177]. C3G and cyanidin-3-rutinoside are potent inhibitors of the initiation events of esophageal cancer due to their ability to impact the metabolic activation and detoxification of NMBA [205]. Moreover, F344 rats with a diet supplemented with lyophilized anthocyanins (5–10% g/kg) show reduced formation of NMBA-induced O^6^-methylguanine adducts in esophageal DNA, providing evidence that consumption of berries influences the metabolism of NMBA, leading to reduced DNA damage [206]. Bio-fractionate studies reveal that diets containing approximately the same quantity of anthocyanins, regardless of their source, have a similar anti-carcinogenesis effect on esophageal cancers [178]. In line with these observations, feeding of anthocyanins extracted from seven types of berries is capable of inhibiting the initiation and progression of NMBA-induced tumors in F344 rats [179]. Furthermore, diets supplemented with 6.1% black raspberry powder, an anthocyanin-rich fraction of black raspberries (0.8 mg/g), or 500 μg/mL PCA had similar effects on cytokines produced in the esophagus and circulating in the plasma of NMBA-treated rats; relative to the NMBA-only control, proinflammatory IL-1β expression was decreased while IL-10 and IL-12 expression increased [180]. However, a crude black raspberry-supplemented diet was more effective in reducing inflammation and NMBA-induced carcinogenesis in F344 rats than the anthocyanin metabolite, PCA [207], suggesting additive or synergistic effects by the components of the crude extract. Recently, synthetic analogs of anthocyanins have been produced and tested. Dracorhodin perchlorate (DP), a synthetic analog of the anthocyanin red pigment dracorhodin, exerts various pharmacological effects, including anti-cancer activity in human ESCC cells due to G_2_/M phase cell cycle arrest through upregulation of p21 and p27, and downregulation of cyclin B1 and Cdc2 [208]. Importantly, anthocyanins are superior to the combination of celecoxib, a selective COX-2 inhibitor, and S,S’-1,4-phenylene-bis(1,2-ethanediyl)bis-isothiourea (PBIT), a selective iNOS inhibitor, in suppressing carcinogen-induced ESCC in rats [209].

### 5.3. Gastric Cancer

Gastric cancer is a heterogeneous malignancy that is mostly induced by *H. pylori* infection and is ranked as the 4th highest cause of cancer-related deaths [210]. Evidence that anthocyanins are effective in gastric cancer prevention is limited. A recent meta-analysis of cohort and case-control studies revealed that there is no significant association between anthocyanin intake and gastric cancer risk, nor is there any dose-dependent relationship [211]. In contrast, a case-control study of 334 gastric cancer patients showed a positive correlation between consumption of anthocyanins and reduced incidence of gastric cancer; the positive effects were predominantly seen in women [212]. Interestingly, anthocyanins are potent inhibitors of the biogenesis of *H*. *pylori* virulence proteins [213], suggesting a possible suppressive effect on *H*. *pylori* infections. Furthermore, anthocyanins extracted from black soybean inhibited *H. pylori*-induced inflammation in gastric cells (AGS) while reducing ROS, iNOS, and COX-2 expression, as well as proinflammatory IL-8 production [181]. Similarly, mulberry anthocyanins suppress the proliferation of SGC-7901 gastric cancer cells and upregulate their expression of caspase-8 and beclin-1, as well as increasing the Bax/Bcl-2 ratio [214]. However, anthocyanidins are more effective than anthocyanins in reducing in vitro growth of gastric cancer cells. For example, malvidin induces apoptosis of AGS cells by causing G_0_/G_1_ phase cell cycle arrest more effectively than its glycosidic form [157]. Anthocyanins also enhance the anti-cancer effects of chemotherapeutic drugs. In this regard, Lu et al. [215] have demonstrated an additive anti-cancer effect of anthocyanins in combination with cisplatin. Nevertheless, evidence of the role of anthocyanins in gastric cancer prevention is limited, indicating the need for further investigation.

### 5.4. Liver Cancer

Liver cancers are primarily comprised of hepatocellular carcinoma (HCC) and cholangiocarcinomas mixed liver carcinoma, of which HCC is the most common [216]. The major risk factors for HCC are chronic hepatitis B and C virus infections, cirrhosis, and metabolic liver disease [217]. Dietary interventions for the prevention of hepatic carcinogenesis have been studied for decades, and a possible role of anthocyanins in liver cancer prevention has been investigated. Anthocyanins from haskap berry (*Lonicera caerulea*) cv. Beilei are beneficial in adjusting the redox balance of human SMMC-7721 HHC cells in vitro, as well as promoting anti-tumor immune responses in mice bearing H22 hepatoma tumors [218]. These anthocyanins are also potent blockers of the cell cycle in the G_2_/M phase in hepatocellular carcinoma while at the same time decreasing the level of lipid peroxidation [218]. Malvidin-3-galactoside extracted from blueberry has anti-proliferative effects and induces apoptosis in human HepG2 cells via dose-dependent regulation of cyclin D1, cyclin B, cyclin E, caspase 3, cleaved caspase-3, Bax, and p38 MAPK expression [182]. The anti-invasive properties of anthocyanin exerted on human hepatoma Hep3B cells are the result of downregulating the expression of matrix metalloproteinase (MMP)-2 and MMP-9 [183], suggesting possible anti-metastatic activity. In tert-butyl hydroperoxide (TBHP)-treated human hepatoma cells, pre-treatment with delphinidin, cyanidin, and their glycoside and rutinoside derivatives attenuated DNA single-strand break formation, lipid peroxidation, and redox state alterations [184]. The anti-proliferative effects of anthocyanidins on hepatic carcinoma cells is more pronounced than that of anthocyanins. For example, cyanidin-3-rutinoside showed a prominent inhibitory effect on the growth of HepG2 cells that was not equaled by delphinidin and cyanidin [187]. In a diethylnitrosamine-induced hepatic carcinogenesis rat model, an anthocyanins-rich black currant extract exerted anti-inflammatory effects by increasing the hepatic expression of heat shock proteins and COX-2 in a dose-dependent manner [219]. However, in an aflatoxin-induced hepatic carcinogenesis model, anthocyanins from purple rice bran failed to affect micronucleus formation or xenobiotic-metabolizing enzymes in rat liver [220]. Anthocyanins from different sources may, therefore, not be equally effective against liver cancers.

### 5.5. Colorectal Cancer

Colorectal cancer (CRC) is characterized by the formation of polyps on the inner lining of the colon or rectum and is the 3rd most common cause of cancer-related deaths [221]. The five-year survivability of CRC in the United States is 64%. However, due to a lack of screening programs in many countries, only about 39% of colorectal cancers are diagnosed at an early stage, leading the majority being detected at a later stage, probably after metastasis has occurred [222]. Since about 80% of CRCs have a sporadic origin, it may be possible that adopting beneficial dietary and lifestyle practices could prevent CRC [223]. A recent meta-analysis of seven different studies revealed an inverse association between total anthocyanin consumption and CRC risk, although a dose-response relationship was not found [76]. Similarly, a recent systemic review elaborated on the positive linkage between the anthocyanin intake and reduced CRC risk via interference with CRC cell signaling and proliferation, as well as the ability to induce apoptosis by effects on several molecular pathways [224]. The protective effects of anthocyanins, crude berry extracts, and fruits with vivid purple and blue shades upon CRC have been well documented by in vitro and in vivo studies of colon cancer and inflammation models [140]. In many cases, anthocyanins have been administered in pure forms or as part of the whole fruit following processing by freeze-drying. Many of the chemopreventive properties observed seem to occur through inhibition of signaling pathways known to be important in the pathogenesis of CRC. For example, treatment of Colo 320DM cells with purple-shoot tea extracts resulted in reduced cell proliferation due to the blockade of cell cycle progression during the G_0_/G_1_ phase, as well as the induction of apoptotic death [144]. Aberrant expression of micro RNA, a class of small, endogenous, non-coding, single-stranded RNAs that bind to the 3′-untranslated region (3′-UTR) complementary sequences of their target mRNA, plays a critical role in the initiation, promotion, and progression of CRC [225]. Numerous studies have shown that anthocyanins prevent the development of CRC by improving miRNA regulation [226,227]. Exposure to black raspberry anthocyanins results in the overexpression of miR-24-1-5P in the colon tissue of carcinogen-treated mice, leading to significant suppression of β-catenin that in turn reduced CRC cell proliferation and migration, and enhanced survival [226]. In another study, black raspberry anthocyanins decreased miR-483-3p expression, which is oncogenic in a mouse model of CRC [227]. Colon-available raspberry extract (an extract that mimics the composition present in the colon) has been used to assess its chemopreventive properties in cultures of Caco-2, HT-29 and, HT 115 CRC cells [228]. Once consumed, anthocyanins are gradually digested during passage through the GI tract; thus, the composition of extracts that are available at the colon do not always mimic the composition of the original extract. However, a colon-available extract of raspberry anthocyanin, characterized by increasing amounts of polyphenols and polyphenol breakdown products but less anthocyanin than in the original, was potent in reducing H_2_O_2_-induced DNA damage in HT-29 cells and the proliferation of HT-115 CRC cells but did not affect the membrane integrity of Caco-2 cells [228]. Cancer stem cells are responsible for the initiation and progression of colorectal tumors [229]. It is, therefore, important to note that anthocyanin-containing baked purple-fleshed potato extracts suppressed the proliferation of colon cancer stem cells and increased their death by apoptosis in a p53-independent manner [230]. Furthermore, anthocyanins were found to reduce the levels of the Wnt pathway effector β-catenin, a critical regulator of cancer stem cell proliferation and epithelial-to-mesenchymal transition. Topoisomerase I and II activity in HT-29 CRC cells is also preferentially diminished in the presence of berry anthocyanin via its action as a topoisomerase-inhibiting catalyst [163]. Anthocyanins also modulate TJ proteins; anthocyanin from *Vitis coignetiae Pulliat,* a Korean fruit, increases the transepithelial electrical resistance of HCT 116 cells and suppresses MMP-2 and MMP-9 expression in a dose-dependent manner [231]. These findings indicate that anthocyanins may be able to maintain the integrity of epithelial barriers.

In a bio-fractionate study, anthocyanins exhibited greater anti-proliferative activity (>50%) in HT-29 and Caco-2 cell cultures than non-anthocyanin polyphenols such as flavonol, tannin, and phenolic acid fractions [189]. Moreover, structure-function relationships of anthocyanins from various sources reveal that non-acylated monoglycosylated form of anthocyanin is a more potent inhibitor of HT-29 CRC cell proliferation [232]. The varying compositions and degrees of growth inhibition suggest that the chemical structure of anthocyanins may play an essential role in their cell growth inhibitory activity. In this regard, there is a distinguishable difference between the anti-proliferative activity of anthocyanins extracted from leaf versus those extracted from the tuber of purple sweet potato in cultures of HCT-116 cells [233]. The leaf contains more cyanidin than the roots. The glycosylated form of cyanidin also suppresses the growth of tumor xenografts by targeting T-LAK-cell originated protein kinase, which plays a role in cell cycle regulation and mitotic progression [234]. Compared to delphinidin-3-*O*-glucoside, C3G is better able to activate the immune response in the tumor microenvironment by inhibiting the action of immune cell checkpoints [235]. However, orally administered C3G did not protect against DNA damage in a vitamin E-deficient rat model, although C3G did protect against DNA damage in human colonocytes, decreasing DNA strand breakage by 39% [236].

A significant amount of data derived from in vivo work demonstrates a potential set of benefits from dietary anthocyanins in terms of CRC prevention. As chronic inflammation is an important event leading to colon cancer, a number of pre-clinical trials have investigated the relationship between anthocyanins and acute or chronic colitis with the DSS-induced colitis model being the most common (Table 3). For example, orally administered anthocyanin-rich blueberry extract attenuates the development of DSS-induced experimental colitis in mice by reducing the accumulation of myeloperoxidase and malondialdehyde in the colon and prostaglandin E2 levels in serum while increasing the levels of SOD and catalase compared to untreated mice with colitis [237]. In addition, a diet supplemented with red raspberries resulted in a reduction in the disease activity index, histological damage, and expression of inflammatory mediators while facilitating repair of the epithelium in animals with DSS-induced colitis [238]. Azoxymethane (AOM) is a potent colon carcinogen that is used with/without DSS to induce colitis-associated carcinogenesis or non-colitis-associated carcinogenesis, respectively, in rodents [239]. In AOM-induced carcinogenesis in F344 rats, a diet that was supplemented with lyophilized black raspberries resulted in a dose-dependent reduction in aberrant crypt foci multiplicity [190]. Dietary supplementation with lyophilized strawberries also exerts an anti-cancer effect against inflammation-mediated colon carcinogenesis in mice by reducing the expression of pro-inflammatory mediators, suppressing nitrosative stress, and decreasing phosphorylation of phosphatidylinositol 3-kinase, Akt, extracellular signal-regulated kinase and NF-κB [240]. In addition, a significant decrease in adenoma number was attributed to the consumption of anthocyanin-rich sweet potato by the APC^MIN+/−^ mice [191]. In the same animal model, oral supplementation with freeze-dried black raspberries reduced the number and size of intestinal and colonic polyps [192]. The berry supplement also significantly reversed the production of 23 APC-regulated metabolites, including 13 colonic mucosa, eight liver, and two fecal metabolites that are involved in amino acid, glutathione, lipid, and nucleotide metabolism. These results suggest the metabolic modulatory effects of anthocyanins in APC^MIN+/−^ mice may contribute to the suppression of CRC.

## 6. Epidemiological Studies

Only a limited number of clinical studies have investigated the effect of anthocyanins in GI cancer prevention; however, a positive relationship between anthocyanin intake and reduced risk of GI cancers has been revealed. The consumption of a wide range of anthocyanins and a reduction in the incidence of GI cancer malignancy is associated with the mechanisms involved in (i) improving the intestinal TJ barrier integrity via AMPK activation; (ii) down-regulating pro-inflammatory molecules; (iii) inhibiting redox dysregulation; (iv) inhibiting cell proliferation by initiating cell cycle arrest; and (v) activating apoptotic pathways. More specifically, black raspberry has been extensively studied for its potential to reduce oral intraepithelial neoplasia (OIN) lesions. In a placebo-controlled study, topical application of a mucoadhesive gel containing 10% *w*/*w* freeze-dried black raspberry powder four-times daily for six weeks to OIN lesions significantly decreased lesion size, the severity of oral dysplasia, and loss of heterozygosity indices [248,249]. Elevated levels of COX-2 and iNOS are correlated with the malignant transformation of OIN. Treatment with a black raspberry powder-containing gel uniformly suppressed the expression of genes associated with RNA processing, growth factor signaling, and inhibition of apoptosis in human premalignant oral lesions [250]. Furthermore, the gel application reduced the expression of COX-2 and iNOS, which correlates with malignant transformation of oral intraepithelial neoplasia, while reducing vascular densities in the superficial connective tissues and inducing genes associated with keratinocyte terminal differentiation. In another study, daily intake of black raspberry slurry for six months was assessed in 77 individuals with Barrett’s esophagus; however, the severity of Barrett’s esophagus was not affected [251]. As the transition time of black raspberry anthocyanins through the esophagus is short, lesions may have failed to absorb a sufficient amount of anthocyanins. It is, therefore, essential to prepare anthocyanins in formulas that have an enhanced absorption into the esophageal tissues. Similarly, a cohort study of 469,008 participants was carried out to determine the association between flavonoid (including anthocyanin) intake and esophageal, head and neck, and gastric carcinoma risk by analyzing the flavonoid intake in each food item using the 2015 USDA Expanded Flavonoid Database for the Assessment of Dietary Intakes [252]. Based on the reported data, flavonoid intake has no relationship with the incidence of esophageal or gastric cancer but showed an inverse relationship with head and neck cancer. Clinical studies on humans have provided additional evidence for the use of black raspberry in CRC prevention. Black raspberry supplementation modifies energy-generating pathways by regulating multiple metabolites, which, in turn, aid in CRC prevention [253]. Among 20 patients who received a freeze-dried black raspberry supplement of 1062 mg total anthocyanins/individual/day for nine weeks, the demethylation of tumor suppressor genes was increased [253]. Methylation of tumor suppressor genes causes their silencing and can induce mutational events, which plays a fundamental role in precipitating the development of a large and diverse number of human GI cancers [254]. Based on epidemiological studies, the value of anthocyanins in GI cancer prevention remains controversial and therefore requires additional investigation.

Circulating cytokines are one of the key indicators of risk and stage of CRC; expression of IL-6, IL-8, TNF-α is upregulated, and IL-2 is downregulated in CRC development [255]. Despite the positive results reported in cell-based studies, anthocyanin intake has not always been shown effective in altering the cytokine profile in favor of CRC reduction. For example, a slurry of freeze-dried black raspberry 354 mg/day in 100 mL of drinking water was not effective in modulating the plasma concentrations of cytokines in 24 CRC patients and, indeed, increased the plasma concentrations of granulocyte-macrophage colony-stimulating factor (GM-CSF), which promotes tumorigenesis by stimulating the epithelial cell release of vascular endothelial growth factor (VEGF) that enhances tumor survivability. Those findings reveal controversy surrounding the effectiveness of anthocyanins in CRC prevention [256]. However, on the other hand, DNA methylation, methyltransferase I protein expression and p16 promoter methylation were significantly reduced in 14 FAP patients who received black raspberry powder for nine months by oral administration (1787 mg/individual/day) and rectal insertion (595 mg/individual/day) of two suppositories [257]. Although the tumor burden was reduced, raspberry supplementation did not reduce the number of tumors. Black raspberry supplements are reasonably well tolerated by cancer patients, showing no adverse effects. However, the anti-cancer effect of black raspberry anthocyanin supplementation might be impacted by variables such as the microbiome. In another clinical study, oral supplementation with commercially available black currant extract powder (672 mg/day) altered gut microbial composition in 30 healthy adult male and female subjects by increasing the relative abundance of beneficial bacteria (*Lactobacillus* and *Bifidobacteria*) while reducing *Clostridium* and *Bacteroides* numbers and inhibiting β-glucuronidase [258]. Anthocyanins are, therefore, potent modulators of gut microbial dysbiosis in CRC.

## 7. Conclusions and Future Directions

GI cancers remain the most common reason for cancer-related deaths worldwide. The sporadic nature of the disease provides a rationale for diet-related cancer prevention, as has been supported by considerable evidence generated from in vitro and in vivo studies and clinical trials. In this review, the diverse beneficial effects of anthocyanins in the chemoprevention of GI cancers have been discussed. Anthocyanin-rich extracts and isolated individual anthocyanins in GI cancer prevention have been investigated during the past two decades. Most of the investigated anthocyanin-rich extracts also contain other flavonoids and polyphenols, ascorbic acid, and sugars. Therefore, the chemopreventive properties of anthocyanin-rich extracts are attributed to the respective health-promoting effects of combinations of compounds; however, the synergistic effect of anthocyanins in phytocomplexes needs to be studied. Although the molecular mechanisms of cancer prevention by anthocyanins are not well elucidated, the involvement of anthocyanins in the modulation of MAPK, NF-κB, AMPK, and Wnt/β-catenin pathways of normal and cancer cells are well documented. Dietary anthocyanins contribute to the prevention of GI cancer initiation via their antioxidative properties. Findings over the past decade reveal anthocyanin-mediated direct scavenging of ROS, the elevation of oxygen radical absorbing capacity of normal cells, stimulation of the expression of phase II detoxification enzymes, reduction in the formation of oxidative DNA adducts, and inhibition of mutagenesis by environmental toxins and carcinogens. As a sub-class of flavonoids, anthocyanins may transition from antioxidants to prooxidants depending on the concentration and its micro-environment, such as the presence of transition metal ions. However, we have not come across any report on the prooxidant effect of anthocyanin related to GI cancers. Further, anthocyanins have the potential to reduce microbial dysbiosis and GI tract inflammation by improving intestinal TJ barrier integrity by promoting the mRNA expression of key barrier-forming TJ proteins such as occludin, claudin-5, and zonnula occuldin-1 via upregulating the GLP-2 intestinal hormone levels. Anthocyanins are also potent inhibitors of GI cancer cell growth due to their ability to increase the levels of cyclin-dependent kinase inhibitor proteins and cell cycle regulatory proteins such as p53, p21, and p27, arrest the cell cycle and induce GI cancer cell apoptosis by facilitating the release of mitochondrial cytochrome c, activation of caspase-releasing enzymes and increasing the Bax:Bcl-2 ratio. These factors all contribute to the prevention of GI cancer development. Anthocyanins also inhibit GI cancer progression via inhibiting metastasis by downregulation of MMP-2 and MMP-9 activity, which maintains the integrity of the epithelial barrier. However, it is important to note that many of the documented beneficial effects of anthocyanins are based on cell-based and experimental animal model-based studies. The concentration of anthocyanins with antiproliferative efficacy ranges from 25 to 200 µM in cell cultures, while the low systemic bioavailability of anthocyanins significantly diminishes their in vivo chemopreventive properties. Additional investigation is also required to develop methods of enhancing the bioavailability of anthocyanins. Novel food technologies, such as micro-encapsulation and nano-encapsulation of anthocyanins that might enhance anthocyanin delivery to targeted sites of the GI tract need further study. Identifying the most effective anthocyanin metabolites in terms of chemoprevention will facilitate the design of novel therapeutics for GI cancer prevention and treatment. Given that mixtures of different anthocyanins may be more effective than single compounds in managing the GI cancers, identification of optimal synergistic combinations of anthocyanins, as well as their formulation with other bioactives in GI cancer prevention, is a logical approach. However, as current knowledge regarding anthocyanins in GI cancer prevention is limited, future investigations are necessary to validate laboratory findings using properly designed human dietary intervention studies.

## Figures and Tables

**Table 1:** Hereditary basis of GI cancers.

Type of the Cancer	Syndrome		Associated Germline Mutations	Reference
**Esophageal**	Familial Barrett’s esophagus, Familial esophageal adenocarcinoma		MSR1, ASCC1 and CTHRC1	[29]
	Tylosis with esophageal cancer-squamous cell carcinoma		RHBDF2	[30]
**Gastric**	Diffuse hereditary gastric cancer-adenocarcinoma		CDH1 (E-cadherin)	[31]
**Pancreatic**	Hereditary pancreatitis		PRSS1, CFTR, SPINK1, CTRC	[32]
	Hereditary breast and ovarian cancer		BRCA1/2	
	Peutz-Jeghers syndrome		STK11/LKB1	
	Familial atypical multiple mole melanoma syndrome		CDKN2A/p16	
	Familial adenomatous polyposis		APC	
**Colorectal**	Familial adenomatous polyposis		APC	[33,34]
	Lynch syndrome		EPCAM, MLH1, MSH2, MSH6, PMS2	
	MYH associated polyposis		MUTYH	
	Hamartomatous polyposis syndrome	Peutz-Jeghers syndrome	STK11	
		Juvenile polyposis syndrome	SMAD4, BMPR1A	
	Attenuated Familial adenomatous polyposis		APC	
**Small intestine**	Familial adenomatous polyposis		APC	[35]
	Lynch syndrome		Mutations in mismatch repair genes	
	Juvenile polyposis syndrome		SMAD4	
	Peutz-Jeghers syndrome		STK11	
**Liver**	α-1 antitrypsin deficiency		SERPINA1	[36,37,38,39,40]
	Hereditary hemochromatosis		HFE	
	Hereditary tyrosinemia type 1		FAH	
	Glycogen storage disease type 1		G6PC, SLC37A4	
	Wilson’s disease		ATP7B	
	Niemann-park disease		SMPD1 AND NPC1 OR NPC2	
**Biliary**	Bile salt export pump deficiency		ABCB11	[41]

**Table 2:** Evidence that anthocyanins have chemopreventive properties against GI cancer and their potential cellular mechanisms.

Source of Anthocyanin	Dosage	Cell Line/Animal Model	Observations	Reference
***Oral Cancer***				
**Blueberry and malvidin**	50 µg/mL	Human oral SCC131 cells	Reduced STAT-3 phosphorylation and nuclear translocation Induced cell cycle arrest at G1/S phase and apoptosis	[152]
**Cranberry extracts**	25–200 µg/mL	Human oral epidermal KB, CAL-27 cancer cells	Inhibited cell proliferation	[169]
**Black rice (*Oryza Sativa* L.)**	100–500 µg/mL	Human tongue epithelial CAL 27 cells	Inhibited cell migration and invasion Inhibited activity of MMP-2 Inhibited NF-κB p65 protein expression Suppressed Pl3K/Akt pathway	[170]
**Commercial anthocyanin**	250 µg/mL	Human oral SCC	Reduced cell viability, Inhibited migration, and invasion abilities Increased NLRP3, caspase-1, IL-1β protein expression	[171]
**Grape skin extract**	2.5 mg/kg of body weight	Male Wistar rats; 4-nitroquinoline 1-oxide induced tongue carcinogenesis	Reduced epithelial dysplasia Reduced p-NF-κB p50 and MyD88 protein expression No change in copper-zinc superoxide dismutase, manganese superoxide dismutase, and catalase gene expression	[172]
**Lyophilized strawberry**	5% or 10% *w/w* for 12 weeks	Hamster cheek pouch (HCP) model of oral cancer	Reduced number of tumors Mild and severe dysplasia	[173]
***Esophageal Cancer***				
**Lyophilized black raspberry**	100 μg/mL	Human esophageal microvascular endothelial cells (HEMEC)	Inhibited TNF-α/IL-1β-induced NFκB p65 nuclear translocation, PGE2 production Reduced COX-2, ICAM-1 and VCAM-1 mRNA and protein expression and leukocyte binding Inhibited Akt, MAPK and JNK phosphorylation	[174]
**Lyophilized black raspberry, C3G, C3R**	10–50 µg/mL	RE-149DHD and RE-149 rat esophageal cancer cell lines	Inhibited cell growth Induced apoptosis Reduced COX-2, iNOS mRNA expression	[175]
**Lyophilized black raspberry**	2.5% *w/w* of the diet	Male Sprague-Dawley rats, EDA surgery-induced carcinogenesis	No change in COX-2 level Reduced MnSOD levels Not effective in the prevention of reflux-induced esophageal adenocarcinoma	[176]
**Lyophilized black raspberry**	5% *w/w* for 10 weeks	NMBA-induced carcinogenesis in F344 rats	Influenced the metabolic activation and detoxification of NMBA Reduced cell proliferation, inflammation, and angiogenesis Inhibited *CYP2a2* mRNA expression	[177]
**Lyophilized black raspberry**	5% *w/w* for 30 weeks	NMBA induced carcinogenesis in F344 rats	Reduced NF-κB protein expression Reduced number and volume of NMBA-induced papillomas Inhibited cell proliferation and, inflammation Induced apoptosis	[178]
**Either black or red raspberries, strawberries, blueberries, noni, açaí or wolfberry**	5% *w/w* for 35 weeks	NMBA induced carcinogenesis in F344 rats	Reduced serum cytokines, IL-5, and GRO/KC protein expression No change in serum IL-1ß, IL-4, IL-13, and TNF-α protein expression Increased IFN-γ protein expression	[179]
**Lyophilized black raspberry, anthocyanin extract, PCA**	6.1% *w/w*, 0.35 ppm and 500 ppm respectively	NMBA induced carcinogenesis in F344 rats	Reduced IL-1β protein expression Increased IL-10, IL-12 protein expression Increased infiltration of both macrophages and neutrophils into the esophagus	[180]
***Gastric Cancer***				
**Malvidin**	50–200 µg/mL	Human AGS cells	Induced apoptosis-arrest G_0_/G_1_ phase Loss of mitochondrial membrane potential Increased BAX/Bcl-2 ration and P38 kinase expression Inhibited ERK activity	[157]
**Black soybean anthocyanin**	12.5–50 µg/mL	*H. pylori-*induced inflammation in AGS cells	Reduced *H. pylori*-induced ROS production Inhibited phosphorylation of mitogen-activated protein kinases, translocation of NF-κB, iNOS, Cox-2 mRNA expressions, IL-8 production	[181]
***Liver Cancer***				
**Black currant**	100, 500 mg/kg body weight for 22 weeks	DENA-induced carcinogenesis in rats	Reduced abnormal lipid peroxidation, protein oxidation and expression of iNOS, 3-nitrotyrosine, Nrf-2	[133]
**Malvidin-3-galactoside**	50–200 µg/mL	Human HepG2 cells	Reduced P-AKT level, MMP-2 and, MMP-9 protein expression Induced apoptosis Increased cyclin-D1, B, E, Caspase-3 protein expression	[182]
**Meoru anthocyanin**	400 µg/mL	Human Hep3B cells	Reduced MMP-2, MMP-9 protein expression Activated NF-κB Promoted anti-invasive effects	[183]
**Isolated anthocyanins**	100 or 500 µg/mL	Rat hepatoma cells (MH1C1)-DNA damaged induced by TBHP	Reduced DNA single-strand formation and lipid peroxidation No change in redox state	[184]
**Meoru anthocyanin**	400 µg/mL	Human Hep3B cells	Reduced cell proliferation, invasion Induced mitochondrial dysfunction Reduced Bcl-2, XlAP, ClAP-1, ClAP-2 protein expression	[154]
**Berry anthocyanin**	0.001–0.1 mg/mL	Human HCC cell lines PLC/PRF/5	Increased Bax, cytochrome c, caspase 3 and, elF2-α protein expression Reduced mTOR, Bcl-2 protein expression	[185]
**Delphinidin, cyanidin, and malvidin**	100 µg/mL	Human HepG2 cells	Reduced cell growth Induced apoptosis-internucleosomal DNA fragmentation Increased Bax: Bcl-2 protein expression Activated c-Jun-N-terminal cascade	[186]
**Black currant**	0.125%, 0.625% *w/w* for 22 weeks	DENA-induced carcinogenesis in Sprague-Dawley rats	Increased incidence, total number, multiplicity, size, and volume of preneoplastic hepatic nodules Abnormal cell proliferation Induced apoptosis Increased Bax: Bcl-2 protein expression	[187]
***Colorectal Cancer***				
**Anthocyanin metabolites (gallic acid, 3-*O*-methylgallic acid, and 2,4,6-trihydroxybenzaldehyde**	10–100 µmol/L	Human Caco-2 cells	Reduced cell viability Induced cell cycle arrest at G_0_/G_1_ Increased caspase-3 activation Inhibited transcription factors NF-κB, AP-1, STAT-1, and OCT-1	[22]
**Standardized anthocyanin-rich extract**	50–500 μg/mL	Human Caco-2 cells	Inhibited cell proliferation Caspase-3 activation Induced apoptosis Increased cellular ROS	[188]
**Lyophilized blueberry**	70–100 μg/mL 50–100 μg/mL	Human HT-29 Human Caco-2 cells	Inhibited cell proliferation 2–7 times increased DNA fragmentation Induced apoptosis	[189]
**Lyophilized black raspberries**	0%, 2.5%, 5%, or 10% wt/wt for 33 weeks	AOM-induced carcinogenesis in F344 rats	Reduced ACF, tumor multiplicity, adenocarcinoma multiplicity by the dose-depended manner	[190]
**Purple fleshed sweet potato**	10% *w/w* of potato skin, potato flesh & 0.12% *w/w* anthocyanin-rich extracted for 18 weeks	C57BL/6J-APC^MIN/+^ mice	Reduced adenoma number (0.12% *w/w* anthocyanin-rich extracted more effective)	[191]
**Lyophilized black raspberries**	5% *w/w* for 8 weeks	APC^MIN/+^ mice	Reduced intestinal and colonic polyp number and size Reversed 23 APC-regulated metabolites, including 13 colonic mucosa, 8 liver and 2 fecal metabolites Reduced putrescine and linolenate levels	[192]
**Cocoplum anthocyanin**	1 to 20 μg/mL	TNF-α stimulated Human HT-29 cells, CCD-18Co non-malignant colonic fibroblasts	Inhibited cell proliferation Increased cellular ROS Reduced TNF-α, IL-1β, IL-6, and NF-κB1 mRNA expression	[117]
**Purple-sweet potato anthocyanin**	0–40 μM	Human colonic SW480 cancer cells	Inhibited cell proliferation Cell cycle arrest at G_1_ phase	[193]
**Purple fleshed potato**	10–30 μg/mL	Human HCT-116 and HT-29 cells	Inhibited cell proliferation Induced apoptosis	[194]
**Cyanidin chloride**	0–50 µM	TNF-α stimulated Human HCT116, HT29, and SW620	Suppressed NF-κB signaling Activated the Nrf2 pathway Increased Bax: Bcl-2 protein and mRNA expression Reduced protein and mRNA expression of TNF-α, IL-6, and IL-8	[195]
**Black raspberry powder**	0.5,5,25 μg/mL	Human HCT116, Caco2 and SW480 cells	Increased protein expression of DNMT1 and DNMT3B Reduced mRNA expression of β-catenin Inhibited cell proliferation Induced apoptosis	[196]
**Anthocyanin-rich extract from Hull blackberries**	0–40 μg/mL	Human HT-29 cells	Inhibited cell proliferation Increased release of IL-12	[197]
**Anthocyanin-rich extracts from bilberry, chokeberry, grape**	3.85 g/kg for 4 weeks	AOM-induced carcinogenesis in F344 rats	Reduced ACF, fecal bile acids and, colonic cellular proliferation Reduced COX-2 mRNA expression (bilberry, grape diets)	[198]
**Anthocyanin-rich extracts from bilberry**	10% *w/w* supplementation for 9 weeks	AOM/DSS-induced colitis-associated carcinogenesis in Balb/c mice	Less reduced colon length Less inflammation Less mean tumor number	[199]

Abbreviations used: AKT, protein kinase B; AP-1, activator protein 1; bcl-2, B-cell lymphoma 2; BAX, Bcl-2 associated X; COX-2, cyclooxygenase 2; CIAP-1, cellular inhibitor of apoptosis protein-1; CIAP-2, cellular inhibitor of apoptosis protein-2; DNMT1, DNA (cytosine-5)-methyltransferase 1; DNMT2, DNA (cytosine-5)-methyltransferase 2; elF2-α, eukaryotic initiation factor 2; ERK, extracellular-signal-regulated kinase; GRO/KC, growth related oncogene; CXCL1; IL-4,5,10,12,13,1β, Interleukin-4,5,10,12,13,1β; IFN-γ, interferon γ; iNOS, inducible nitrogen oxide synthase; JAK, Janus kinase; LC3-I, LC3-I, microtubule-associated protein light chain 3; MMP-2,9, matrix metalloproteinase-2,9; MAPK, mitogen-activated protein kinase; MnSOD, manganese superoxide dismutase; MyD88, myeloid differentiation primary response 88; MTOR, mammalian target of rapamycin; NLRP3, NLR family pyrin domain containing 3; NMBA, N-nitroso methylbenzylamine; NF-κB, Nuclear factor κ-light-chain-enhancer of activated B cells; OCT-1, Octamer proteins in humans; PI3K, phosphoinositide 3-kinases; P-NF-κB, phosphorylated nuclear factor κ-light-chain-enhancer of activated B cells; ROS, reactive oxygen species; STAT-1,3, signal transducer and activator of transcription 1,3; XIAP, X-linked inhibitor of apoptosis protein.

**Table 3:** Experimental findings on the effect of anthocyanin-supplementation on the DSS-induced colitis in experimental animals.

Source of Anthocyanin	Dosage	Treatment	Observations	Reference
**Black rice anthocyanin-rich extract**	25, 50, and 100 mg/kg of body weight	8 weeks old female C57BL/6 mice: administration of 3% DSS for 5 consecutive days in drinking water	Reduced DAI and the histological score of colons, myeloperoxidase (MPO) and nitric oxide (NO) levels and, mRNA expression of IL-6, IL-1β, TNF-α, iNOS, and COX-2	[119]
**Malvidin 3-glucoside**	24 mg/kg of feed weight	4–5 weeks old C57BL/6J male mice: 2 cycles (7 days of 2.5% DSS and 14 days of fresh tap water)	Improved histopathological scores mRNA expression of IL-10 Promoted microbial interactions and restored the *Firmicutes*/*Bacteroidetes* ratio repressed by DSS Reduced abundance of *Ruminococcus gnavus*	[167]
**Blueberry extract**	50 mg/kg body weight	Female Balb/C mice: administration of 3% DSS for 1 week in drinking water	Reduced DAI and improved the macroscopic and histological score of colons Reduced myeloperoxidase accumulation and malondialdehyde in the colon Increased prostaglandin E2 level in serum Reduced levels of superoxide dismutase and catalase Reduced mRNA expression of COX-2 and IL-1β in colonic tissue Reduced nuclear translocation of NF-kB	[237]
**Dietary red raspberry**	5% *w/w* of feed weight	Six-week-old male C57BL/6J mice: administration of 2 repeated cycles of 1% DSS (7-d DSS treatment plus 14-d recovery)	Reduced DAI score and histologic damage Reduced expression of inflammatory mediators Facilitated epithelial repair Reduced β-catenin, STAT3 signaling	[238]
**Maqui berry water extract**	50–200 mg/kg of body weight	6 weeks old wild-type C57BL/6 male mice: administration of 3% (*w/v*) DSS for 1 week in drinking water	Reduced protein expression of COX2 and IL-6 in LPS-stimulated RAW 264.7 cells Reduced inflammatory bowel disease index, MDA, NO, i-NOS, COX-2 protein expression in colon tissue Reduced MPO, TNF-α, and IL-1β protein expression in blood serums Increased protein expression of occludin (Dose-dependent manner)	[241]
**Ginseng berry extract**	50 mg/kg of body weight	C57BL/6 mice: administration of 3% DSS for 8 days in drinking water	Reduced DAI score and histologic damage Reduced numbers and inhibited the activation of colon-infiltrating T cells, neutrophils, intestinal CD103−CD11c+ dendritic cells and macrophages	[242]
**Cranberry extract**	1.5% *w/w* of feed weight	6 weeks old male CD-1 mice: 1.5% DSS for 4 cycles (4 days/cycle, with a 7-day recovery after each of the first 3 DSS cycles)	Inhibited reduction in colon length Reduced DAI and histologic score Increased colonic levels of IL-1β, IL-6, and TNF-α proteins Altered the microbial structure of fecal microbiota in mice Reduced DSS-induced decline in α-diversity Increased abundance of *Lactobacillus* and *Bifidobacterium* Reduced abundance of *Sutterella* and *Bilophila*	[243]
**Dried bilberries**	10% *w/w* of feed weight	Balb/c mice: 2.5% DSS for 1 week in drinking water	Reduced DAI and histologic score Reduced secretion of IFN-γ and TNF-α from mesenteric lymph node cells Intestinal inflammation Prevented inflammation-induced apoptosis in colonic epithelial cells	[244]
**C3G**	Intraperitoneal injected with 1ug C3G every 2 days, a total of 3 times	8–12 weeks old C57BL/6 mice: 3.5% DSS for 1 week in drinking water	No change in body weight and colon length Reduced mRNA expression of IL- 6, IL-1β, IL-18, TNF-α, IFN-γ in colons and mesenteric lymph nodes Reduced CCL22 levels and Tregs induction	[245]
**Anthocyanin-rich tea**	0.13 or 0.16 mg/day by gavage	5 weeks old female ICR mice: 3% DSS for 2 weeks in drinking water	Lowered body weight loss, spleen hypertrophy, and shortening of the colon Reduced deteriorations in survival rate, liver function, colon mucosal IL-1β level (mRNA)	[246]
**Purple carrot extract**	5% *w/w* of feed weight	6–7 weeks old C57BL/6 mice: 2% DSS for 1 week in drinking water	Reduced DSS-induced colon shortening and inflammatory cell infiltration Reduced serum levels of TNF-α and IL-6 (protein) Inhibited colonic mRNA expression of iNOS, COX-2	[247]

Abbreviations used: CCL22, C-C motif chemokine ligand 22; COX-2, cyclooxygenase 2; DAI, disease activity index; IL-6, 1β, interleukin-6, 1β; IFN-γ, interferon-γ; iNOS, inducible nitrogen oxide synthase; MDA, malondialdehyde; MPO, myeloperoxidase; NO, nitrogen oxide; NF-κB, nuclear factor κ-light-chain-enhancer of activated B cells; STAT-3, signal transducer and activator of transcription 3; TNL-α, tumor necrosis factor-α.

## Abbreviations

ACF	Aberrant crypt foci
AOM	Azoxymethane
ARE	Antioxidant response element
Bax	B-cell lymphoma-2-like protein 4
Bcl-2	B-cell lymphoma-2
C3G	Cyanidin-3-*O*-glucoside
CDKs	Cyclin-dependent kinases
COX	Cyclooxygenase
CRC	Colorectal cancer
DNA	Deoxyribose nucleic acid
DSS	Dextran sulfate sodium
ESCC	Esophageal squamous cell carcinoma
FAP	Familial adenomatous polyposis
GI	Gastrointestinal
GSH	Glutathione-s-transferase
HCC	Hepatocellular carcinoma
IFN-γ	Interferon-gamma
IIR	Intestinal ischemia-reperfusion
IL	Interleukin
iNOS	Inducible nitric oxide synthase
MAPK	Mitogen-activated protein kinase
MMP	Matrix metalloproteinase
NF-κB	Nuclear factor-kappa B
NO	Nitrogen oxide
Nrf-2	Nuclear factor-E2-related factor-2
OIN	Oral intraepithelial neoplasia
PCA	protocatechuic acid
RNI	Reactive nitrogen intermediates
RNS	Reactive nitrogen species
ROS	Reactive oxygen species
SCC	Squamous cell carcinoma
TJ	Tight junction
TNF -α	Tumor necrosis factor-alpha
ΔΨm	Mitochondrial membrane potential
